# Inline NMR via a Dedicated V-Shaped Sensor

**DOI:** 10.3390/s23052388

**Published:** 2023-02-21

**Authors:** Eric Schmid, Simon Rondeau, Thomas Rudszuck, Hermann Nirschl, Gisela Guthausen

**Affiliations:** 1 Institute of Mechanical Process Engineering and Mechanics, Karlsruhe Institute of Technology, 76131 Karlsruhe, Germany; 2 Engler-Bunte-Institut, Chair of Water Chemistry and Water Technology, Karlsruhe Institute of Technology, 76131 Karlsruhe, Germany

**Keywords:** low field NMR, inline process monitoring, relaxation, diffusion, NMR-sensor

## Abstract

Process monitoring and control require dedicated and reliable measures which reflect the status of the process under investigation. Although nuclear magnetic resonance is known to be a versatile analytical technique, it is only seldomly found in process monitoring. Single-sided nuclear magnetic resonance is one well known approach for being applied in process monitoring. The dedicated V-sensor is a recent approach that allows the inline investigation of materials in a pipe non-destructively and non-invasively. An open geometry of the radiofrequency unit is realized using a tailored coil, enabling the sensor to be applied for manifold mobile applications in in-line process monitoring. Stationary liquids were measured, and their properties were integrally quantified as the basis for successful process monitoring. The sensor, in its inline version, is presented along with its characteristics. An exemplary field of application is battery production in terms of anode slurries; thus, the first results on graphite slurries will demonstrate the added value of the sensor in process monitoring.

## 1. Introduction

Nuclear Magnetic Resonance (NMR) is well known in academia and industrial research in different experimental and instrumental designs. Two prominent examples are NMR spectroscopy, revealing information about chemical structures, and magnetic resonance tomography (MRT) in medical diagnostics. In industrial production with usually harsher environmental conditions, it is often used as at-line analytics in the form of low field (LF-)NMR, in the food and pharmaceutical industry, for example [1,2]. LF-NMR relies on permanent magnets and is therefore restricted by the magnetic remanence leading to magnetic fields/^1^H Larmor frequencies, currently up to roughly 2.5 T/100 MHz [3]. Compared to high field NMR with ^1^H Larmor frequencies commercially up to currently 1.2 GHz, the signals and the sensitivity are low as the population difference given by the Boltzmann law is significantly smaller. However, the instruments are much smaller and significantly more robust, which is essential for inline process monitoring.

Diverse parameters are known in NMR and also in LF-NMR. A multitude of contributions by a large number of researchers is known in the literature, and on development and applications of LF-NMR. A short and incomplete overview gives an impression about the diversity of the applied NMR methods: spectroscopy in chemical reaction monitoring and references therein [4,5,6,7,8]. Apart from spectroscopy, NMR relaxation reveals an insight into the materials studied as the measures rely on molecular dynamics. This kind of low-field NMR is prominently known in polymer research and applications, irrespective of whether it is measured integrally or spatially resolved, e.g., [9,10]. As the transverse relaxation rate, *R*_2_, is sensitive to low frequency fluctuations mainly within molecules, and as these fluctuations change upon chemical reaction and aging of macro-molecules, LF-NMR, relying on relaxation properties, was often applied in this context. Remarkable inventions were made on the basis of permanent magnets regarding the applicability of NMR in the processes in biological context [11], as well as in process monitoring in harsher industrial environments and quality control, namely by the use of single-sided NMR [12,13,14]. In the present context, questions are addressed as to whether an open structure of an NMR sensor can be realized for process monitoring: Can material-characterizing properties be measured during flow, and is the sensitivity large enough to reliably control a process? What are the constraints concerning the pipe, its geometry and the flow rates? How can NMR sensitivity be optimized? An important prerequisite is that the sensor works completely non-invasively and non-destructively. Second, the pipe must not be opened for mounting the sensor. These prerequisites require dedicated hardware, which was addressed by simulation and technical realization. Finally, an example will be given which shows the usability of the sensor in a current application to batteries anode slurries.

## 2. Materials and Methods

The radio frequency (rf) probe was reconstructed for the V-shaped NMR-sensor [15] to allow for a mobile application in the context of monitoring the properties of a highly viscous, but flowing material. So far, the rf probe of the V-shaped sensor was realized in the conventional closed shape: the rf tank circuit was constructed by two trimmable capacitors and a solenoidal coil. Two regions of the magnet were used for NMR, one small cylindrical volume with moderate magnetic field gradient *G* of 0.35 T/m with a sample diameter of 12 mm, and the other larger volume in the outer part of the magnet with a sample diameter of 42 mm and a much larger magnetic field gradient up to 7 T/m, where the distribution of *G* within the sample volume is not negligible. Please note that the NMR signal is thus inherently sensitive to relaxation phenomena in the sample as well as to diffusion and convection, i.e., motional displacements. These facts were and are explored in single-sided NMR and other dedicated NMR sensors, e.g., [16]. The two places within the magnet were motivated by the application in lubricant quality control, requiring the compatibility with other established analytics concerning sample handling. The NMR-relevant properties of the two cylindrical regions in the V-shaped magnet needed to be considered in addition to the mentioned static magnetic field and its gradient, the rf field amplitude, its homogeneity, and the quality factor of the tank circuit which determines sensitivity and receiver dead time. 

The off-line version, which was mainly used for lubricant quality control, is so far not well suited for inline process monitoring when postulating that the sensor should be removable and mobile without touching the process’ material stream. Thus and primarily, the rf circuit needed a reconstruction and adaptation to these special requirements, which is the main topic of this paper (Section 3 and Section 4), whereby the rf circuit provides the rf field for spin manipulation according to the NMR sequences.

**Figure 1:**
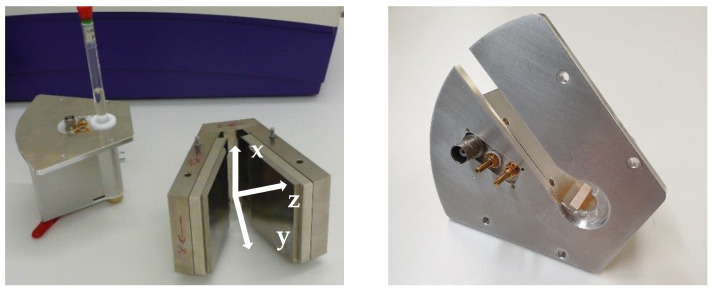
(**Left**): V-shaped NMR sensor (V-magnet with coordinate system and rf probe for off-line measurements) [15] and, on the (**Right**), the inline-capable rf probe. The probe is positioned in the magnets ”V“ so that the sample is aligned along *x* ((**Left**): typical 10 mm sample as used in low-field NMR). Since the inline probe ((**Right**) picture) has a slit, it can be easily mounted on tubes without opening the fluidic flow system. The rf connectors and the trim capacitors are also shown ((**left**): Reprinted/adapted with permission from Ref. [15]).

The coil geometries (Section 3 and Section 4) were simulated and characterized regarding their NMR properties: Larmor frequency *ν_L_*(***r***) and the NMR-intensity *I*(***r***) as function of the spatial coordinates within the sensitive area. The distance of the sample to be measured from the surface coil along *y* (Figure 1) must be mentioned. These parameters were measured using dedicated samples: A reference sample consisting of 20%*w*/*w* H_2_O, 80%*w*/*w* D_2_O and 0.1%*w*/*w* CuSO_4,_ and a graphite slurry for the production of anodes for lithium ion batteries (48.5%*w*/*w* graphite, 1%*w*/*w* carboxy methyl cellulose (CMC) and 0.5%*w*/*w* carbon black).

Methodical aspects are well known from single-sided NMR [17,18,19,20,21,22,23] and are applicable in the present form of the NMR V-sensor. Relaxation and diffusion/fluidic flow need to be considered in the magnetic field with its inherent static field gradients. These static magnetic field gradients provide the sensitivity towards molecular displacements, irrespective of whether they are due to statistical Brownian motion, of turbulent or coherent flow. Most often, multi echo sequences, also in the two-dimensional version of correlated experiments, are explored in which they allow the detection of mainly, but not exclusively, transverse magnetization decays [12,21,23,24,25].

In a first approach, transverse magnetization decays are modelled by an exponential decay function, which is motivated by the basic Bloch equations of NMR, e.g., [26,27]. In the present case, neither the substances are simple liquids nor the technical equipment is ideal in the sense of NMR theory. Often the numerical approach of the inverse Laplace transform is applied in the literature, leading to an effective transverse relaxation time distribution [28,29,30]. It is well known that this approach is limited by the unavoidable experimental noise in the data, and further quantification is hampered by the fact that the distributions are complicated and by no means monomodal. A mathematical-analytical approach is also suitable. The Gamma distribution function was applied in previous works, e.g., on oils, and shows highly reproducible and physically interpretable results [31]. This approach considers that the magnetization relaxation is influenced by the distributions of material properties (disperse systems) and of technical parameters such as the magnetic field gradient, and the spatially varying flip angle of the rf pulses.

## 3. Results

### 3.1. Enabling Inline NMR-Measurements via the V-Shaped Sensor

A solenoidal coil geometry is most commonly used in LF-NMR devices because of the high signal-to-noise ratios compared to other coil geometries, and its suitability regarding the orientation of the magnetic fields. Further advantages concern the beneficial filling factor and the rf homogeneity. Despite this performance, a solenoidal coil is not well-suited for the inline-capable, mobile probe. These special requirements make a surface coil necessary to be mounted into the V-shaped magnet, which can then be flexibly positioned on an existing closed pipe. The loss of the signal-to-noise ratio using a surface coil leads to the motivation to optimize the coil geometry and its position.

Examples for the use of surface coils in low field NMR applications can be found in the literature. For example, McDonald et al. [32] developed the GARField magnet for sub-surface measurements in cement-based materials. Blümich et al. [12] used a surface coil for single-sided NMR with the MOUSE.

The approach in this work is to design a dedicated surface coil that fits the prerequisites of the addressed application. The coil is adapted to the geometry of a cylindrical pipe with a diameter of 10 mm. A bent figure-8 coil was developed and adapted to the intended application. It was compared to a spirally wound, bent surface coil.

#### 3.1.1. Reconstruction of the RF Probe

The V-shaped magnet unit described in [12] was equipped with a closed probe for samples with a diameter up to 12 mm, measuring the samples volumetrically (Figure 1, left). The inline-capable probe now exhibits a characteristic slit (Figure 1, right), through which a tube can be placed near the surface coil without touching the process stream in any form. The cover plate, made of aluminum, provides holes for the two match and tune trim capacitors and the rf connection to a commercial electronics unit (Bruker “the minispec” NF series). There are four holes to fix the probe to the magnet unit on a very defined position. The measuring volume is in the front area in analogy to the offline probe [12]. A conductive box is mounted directly under the cover plate, in which the match and tune trim capacitors and the wirings are placed. The purpose of the box is an efficient shielding of the environmental electromagnetic noise from the rf circuit and to decouple the magnet from the rf circuit. The magnet unit with the inserted probe has the following geometric dimensions: length: 11 cm; width: 13 cm; and height: 8 cm.

The rf circuit consists of two trim capacitors (Voltronics NMTM120CE) and the rf coil. The same capacitors were used for a comparison of the different coil geometries, and only the coil was exchanged. The different surface coils were mounted on a half-shell of the outer housing with rubber spacers for a defined and fixed positioning. The whole probe is designed for easy, subsequent adjustments and optimizations, including a change of the surface coil.

#### 3.1.2. From Volume Coil to Surface Coil: Simulation of the RF Magnetic Field

Simulations of the RF fields, more specific to its magnetic part ***B***_1_, of the two different coil geometries, were carried out in order to define an optimized coil geometry concerning the signal intensity and measuring depth. A spiral wound, bent surface coil was compared to a bent figure-8 coil. The software COMSOL Multiphysics, Version 6.0, was used for the simulations. The coil geometries were set as homogeneous multiturn coils to reduce the simulation’s computational costs. Thus, the current distribution within each winding of the inductor was neglected. The calculated absolute values of the amplitudes of magnetic flux density |***B***_1_(***r***)| are physically not exact, but its spatial profile and distribution are nevertheless relevant and reliable. The modelling of each and every turn of the coils could thus be avoided; nevertheless, the number of turns is considered by a specific parameter. To increase the reliability of the results related to the spatial distribution of the magnetic flux density, the actual value of the current through the coil was not implemented. Instead, the excitation current was set to an arbitrary value of 4 A.

**Figure 2:**
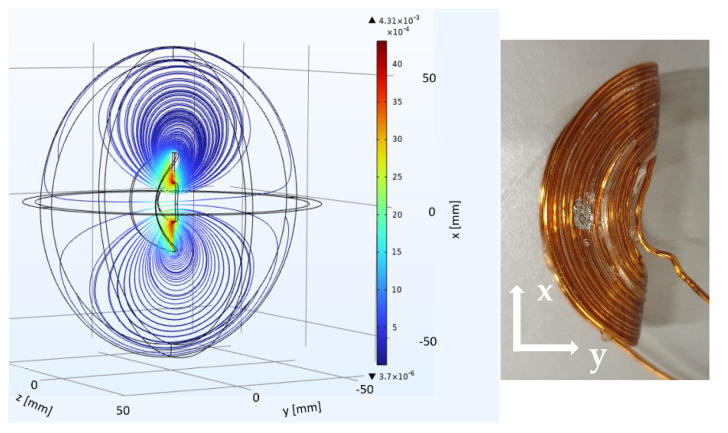
(**Left**): Simulation of the absolute value of the RF-field |***B***_1_(***r***)| of the homogenized version of the spirally wound, bent surface coil. The coils’ geometry is indicated by the inner black lines, while |***B***_1_(***r***)| is color encoded along the scale from blue (low values) to red (high values) and shown along the three spatial coordinates. (**Right**): A picture of the coil with the coordinate system. The sample is aligned along *x* and positioned cantric to the coil.

**Figure 3:**
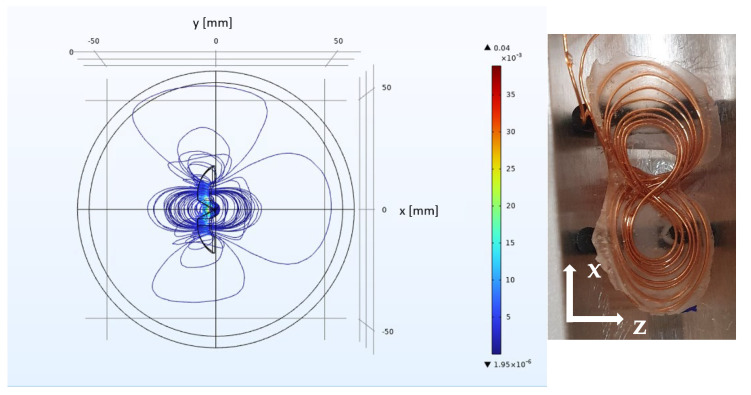
(**Left**): Simulation of |***B***_1_(***r***)| of the homogenized bent figure-8 coil. The coils geometry is indicated by the inner black lines, while |***B***_1_(***r***)| again is color encoded. The field plot shows two condensation areas at both openings of the coil. (**Right**): A picture of the figure-8 coil with the coordinates *x* and *z*. The sample is positioned along *z*, centric to the middle of the coil. The long axis of the cylindrical samples or tubes is aligned along *x*.

**Figure 4:**
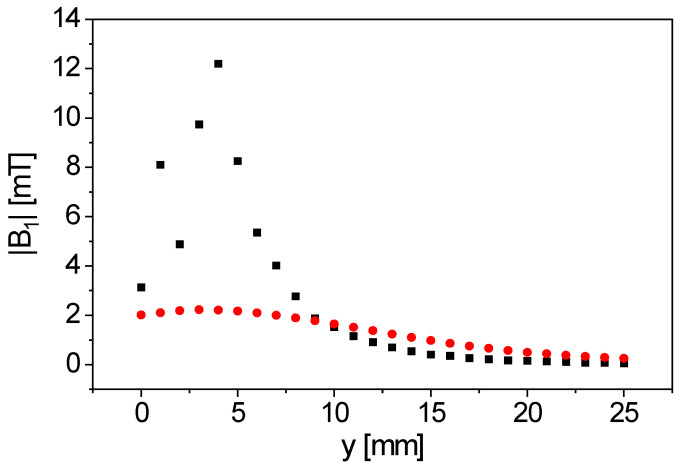
Simulated |***B***_1_| along *y* for the two coil geometries (■: figure-8 coil, ●: spirally wound, bent surface coil). |***B***_1_| of the figure-8 coil is larger than for the spirally wound, bent surface coil up to a distance of 9 mm, leading to an improved NMR sensitivity.

The simulation of the spirally wound, bent surface coil (Figure 2) shows a condensation of the field lines along *x* above and below the opening of the coil. Two condensation areas of the field lines appear for the figure-8 coil (Figure 3) at both openings of the coil. The simulated *y*-profile (Figure 4) shows that |***B***_1_| is considerably larger for the figure-8 coil than for the spirally wound, bent coil, up to a distance from the coil’s surface of 9 mm.

#### 3.1.3. From Volume Coil to Surface Coil: Technical Realization of the Bent Surface Coils

During the manufacturing of a bent surface coil, the wire cannot be wound around a cylindrical carrier similar to how it is performed for solenoidal volume coils. Thus, the insulated copper wire was positioned on an adhesive film, and the wire was wound turn-by-turn as a flat geometry. Two-component, thermo-reversible epoxy glue, which does not lead to a significant background signal, was applied to permanently connect the windings. The glued coil was then bent to the desired shape after heat-softening the epoxy film. After cooling, the bent coil retains its geometry permanently which is then adapted to the cylindrical shape of a 10 mm tube containing a material stream. The motivation for bending was the expected larger filling factor and a more homogeneous ***B***_1_ field.

#### 3.1.4. Comparison of the Coils: Technical Parameters and NMR Properties

Technical and NMR parameters are summarized for three different types of rf coils (Table 1). The solenoidal volume coil was previously used [12]. A spirally wound, bent surface coil is thus compared to the solenoidal volume coil and the bent figure-8 surface coil.

The NMR characteristics (Table 1: pulse duration, receiver gain, profile, etc.) were measured via the Hahn-echo sequence at a small echo time without an increment of *τ_e_* [33]. Thus, the signal intensity was measured step-by-step to determine the profile along *x* and *y*. Care was taken to use samples with sufficiently small dimensions along the coordinates, along which the profiles were investigated. The different coil geometries influence parameters such as the flip angle, the receiver dead time (RDT), or the necessary receiver gain. RDT plays a significant role in selecting the planned application area of the sensor. Graphite slurries already exhibit short transverse relaxation times.

**Figure 5:**
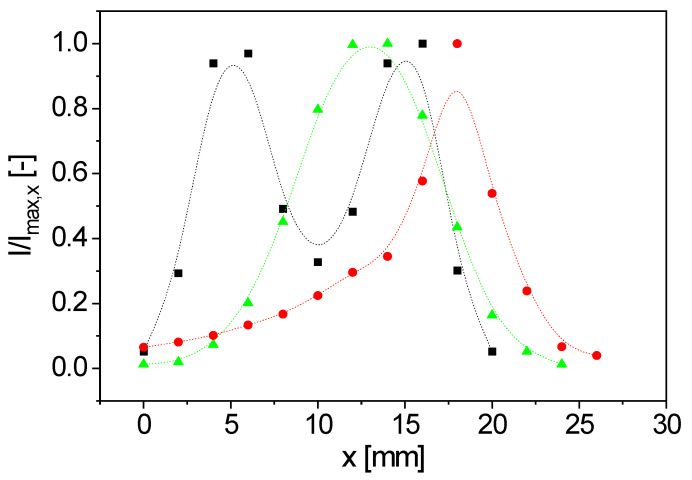
Measured normalized signal intensity profiles along *x* with a small water sample for the three different coil geometries (■: figure-8 coil, *ν_L_* = 22.18 MHz; ●: spirally wound, bent surface coil, *ν_L_* = 21.99 MHz; ▲: solenoidal coil, *ν_L_* = 22.0 MHz). The sample was moved in 2 mm incremental steps along *x* (coordinate system of Figure 1). Pulse duration and frequency were kept constant for each coil. The geometries exhibit different intensity profiles along *x*. The solenoidal coils and the spirally wound, bent surface coils show a single intensity maximum, while two maxima were measured for the bent figure-8 coil, with approximately the same normalized signal intensity.

An important characteristic of an NMR probe is its sensitive volume. The normalized intensity *I*/*I*_max, x_ is therefore compared for the three types of coils. The Hahn-echo sequence was used at ν_L_ = 22.18 MHz to determine the signal intensity as a function of *x* (Figure 5). *I*_max, x_ is the maximal signal intensity over *x* observed for each specific coil.

These *x*-intensity profiles (Figure 5) were measured with a 5 mm NMR tube (filling level 2 mm) with globally determined pulse durations. The sample, with its small filling level, was stepped through the magnet along *x* while the NMR signal intensity of a Hahn echo was measured at the indicated positions. Concerning the *y* and *z* positions: the sample was positioned directly on the surface of the surface coils without touching them and in the center of the volume coil, respectively. Two regions of maximal signal intensity were found near the two openings of the figure-8 coil (Figure 5). ***B***_0_ of the permanent magnet depends on *x*, due to the design of the V-shaped magnet, as does ν_L_(***r***), consequently. In the realized optimal coil position, the same Larmor frequency is observed at the lower and the upper half of the figure-8 coil, which is reflected in the almost identical signal intensities at *x* = 6 mm, and *x* = 16 mm (Figure 5) at ν_L_ = 22.18 MHz. The two maxima in Figure 5, however, are unique for the figure-8 coil, and correspond to the two maxima in |***B***_1_(***r***)|, as evident from the simulation (Figure 3).

**Figure 6:**
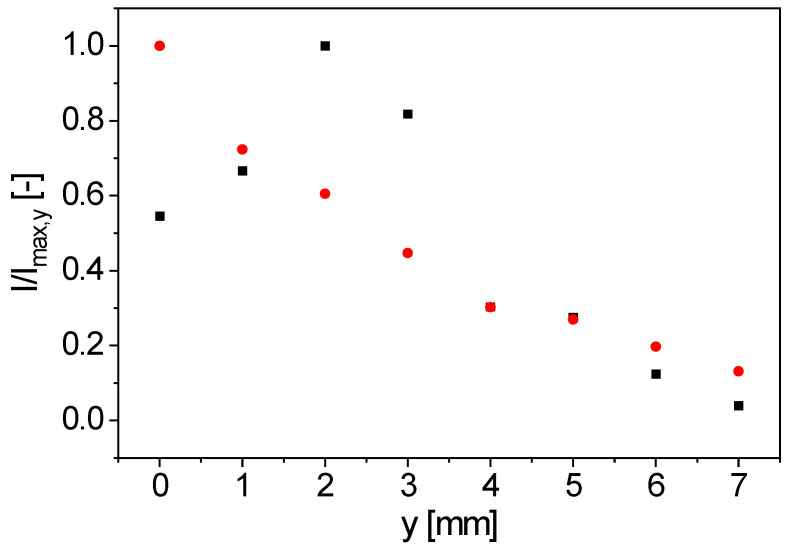
*y*-profiles of the normalized signal intensity for the bent figure-8 coil (■) and the spirally wound, bent surface coil (●), measured with a thin rubber sample. The maximal signal intensity for the spirally wound, bent surface coil is near the surface of the coil, whereas the maximum for the bent figure-8 coil is shifted by around 2 mm in the *y*-direction.

The *y*-profile of signal intensity was measured with rubber on a 10 mm NMR tube. On its outside, a rubber sheet (thickness 1 mm) was glued to provide a relatively thin sample for measuring the distance profile (Figure 6). *y* = 0 mm is therefore defined directly on the surface of the coils. The measurements show that the maximal signal intensity is not directly at the surface of the bent figure-8 coil, but at a distance of 2 mm, in agreement with the ***B***_1_ simulation (Figure 4). The sample was detectable up to a distance of *y* = 7 mm. This implies that not the entire volume of a sample with a diameter of 10 mm is detected. The position of the maximal signal intensity at a distance of 2 mm from the coil surface means that the most sensitive area of the inline-capable probe is in the sample itself when using tubes with a wall thickness smaller than 2 mm. For comparison, the realized sample tube for inline measurements has a wall thickness of 1 mm.

### 3.2. NMR-Measurements on Disperse Suspensions

**Figure 7:**
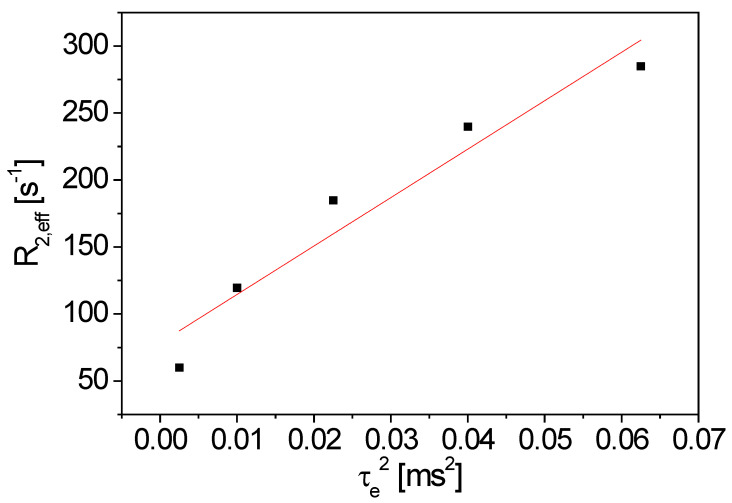
*R*_2,eff_ as a function of the squared echo time *τ_e_*^2^ for an anode slurry of 48.5%*w*/*w* graphite, 1%*w*/*w* CMC and 0.5%*w*/*w* carbon black. The red line evidently shows the deviation from pure transverse relaxation and diffusion contributions.

**Figure 8:**
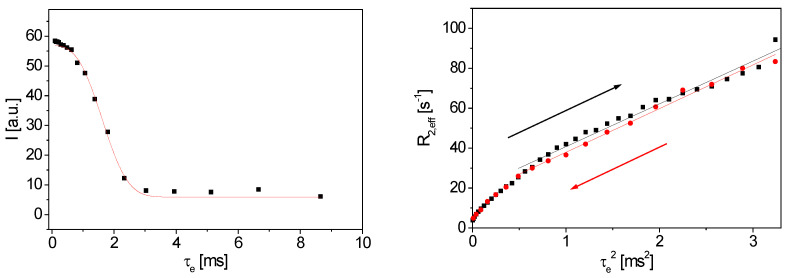
(**Left**): Measurements on the reference sample. Hahn-echo decay was used to determine the static gradient near the maximum sensitivity of the bent figure-8 coil (*G* = 1.14 T/m). Modelling of the magnetization decays includes an offset. (**Right**): A deviation from the expected linear behavior of *R*_2,eff_(*τ_e_*^2^) is found for a small *τ_e_* due to sample heating. At large *τ_e_* and a reduced sample filling level (red dots), RF heating is less significant, and the range of linearity is larger.

An aqueous, extruded anode slurry was analyzed with a solid content of 50%*w*/*w* for the anode manufacturing of lithium-ion batteries (48.5%*w*/*w* graphite, 1%*w*/*w* CMC, 0.5%*w*/*w* carbon black). The sample temperature was 25 °C, and the long axis of the 10 mm sample tube parallel to *x* was along the gravity direction. The filling level was 10 cm to guarantee a good sample homogeneity in the sensitive volume. The results were obtained with the figure-8 coil. The CPMG pulse sequence (Carr-Purcell-Meiboom-Gill) [34,35] was used with 3000 echoes, while *τ_e_* was incremented. The repetition time was 2 s. It is well known that the transverse magnetization decay depends on the magnetic field gradient, leading to a sensitivity towards diffusion phenomena [34,35]. Please note that the magnetic field ***B***_0_ of the V-shaped sensor shows magnetic field gradients in all spatial dimensions, similar to the facts in other dedicated NMR sensors [32,36,37]. Therefore, only the average magnetic field gradient contributes in the measurements, which depends on the sample volume and the position of the RF coil. The signal decays were modeled by a mono-exponential function with sufficient numerical accuracy, leading to the effective transverse relaxation rate *R*_2,eff_. *R*_2,eff_ depends on *τ_e_* (Figure 7, [23]), as expected. However, an effective diffusion coefficient in the slurry cannot be calculated via linear regression. The echo times *τ_e_* are relatively small, so that, among other factors, sample heating and consequently, convection occur. Both relaxation and diffusion depend on the sample temperature. The RF energy intake leads to an increase of the temperature for small *τ_e_*, in particular. The graphite particles in the slurry may additionally act as energy absorbers. In principle and in summarizing, measurements at a larger *τ_e_* would be required. Additional measurements were performed with a reference sample to prove this data interpretation hypothesis.

The reference sample (20% H_2_O, 80% D_2_O and 0.1% CuSO_4_) was filled into a completely filled 5 mm NMR tube. The average static gradient was determined via the Hahn echo sequence (increment factor for τ_e_: 1.3; recycle delay: 10 s) to *G* = 1.05 T/m with *D* = 2.4∙10^−9^ m^2^/s at 27 °C and *R*_2_ = 2 s^−1^ (Figure 8 left). Deviations between the measured points and the modeling are due to experimental noise (magnitude data) and the inherent distribution of *G*.

In a first CPMG measurement on the reference sample, *τ_e_* was varied in the range [0.05, 1.8] ms with a recycle delay of 4 s (Figure 8, right, black dots). A deviation from the expected linear relation *R*_2,eff_ (*τ_e_^2^*) was observed for a small *τ_e_*, which was also in this case of measurements on the reference sample. To further test the hypothesis of sample heating and associated convection, the experiment was repeated. The repetition time was extended to 40 s to ensure thermostating of the sample. The sample filling height was reduced to 1 cm, and τ_e_ was reduced, in addition to comparing to the first measurement (Figure 8, right, red dots). As a result, the linear range is extended over a significantly larger range of *τ_e_*, and the calculation of the diffusion coefficient with *G* = 1.14 T/m results in *D* = 2.9∙10^−9^ m^2^/s, which is within the expectation, considering the distribution of *G* and the sample heating.

Please note that the significant difference in *R*_2,eff_ of the two samples, the reference sample (Figure 8) and the graphite slurry (Figure 7). In contrast to the reference sample, *τ_e_* was one order of magnitude smaller (*τ_e_* ϵ [0.05, 0.25] ms) in the case of the graphite slurry, since *R*_2,eff_ is much larger.

Diffusion is expected to be hindered in the slurry, leading to a reduction, i.e., a flatter dependence on *τ_e_*, while *R*_2_ is enhanced by paramagnetic relaxation enhancement due to nanoscale Fe and Fe oxides in typical concentrations up to 50 ppm in the used graphite powder.

**Figure 9:**
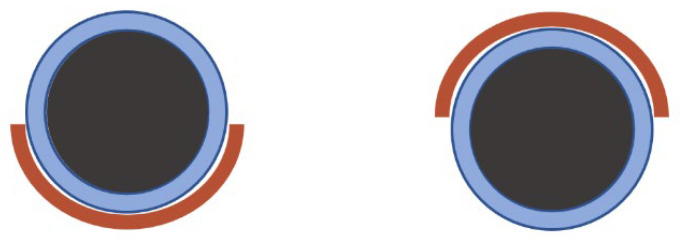
Positioning of the surface coil on the bottom (**left**) and the top (**right**) of the horizontally fixed 10 mm pipe.

Coming back to the potential application of the inline capable V-sensor, an important question concerns sedimentation which would influence the subsequent processing steps of coating, calendaring, and drying. The V-shaped sensor equipped with the figure-8 surface coil can be easily used to investigate the status of a slurry in a pipe. Positioning the magnet such that the gravity axis is along *y* (Figure 1) allows the investigation of the pipe’s interior, which is, with its long axis, also perpendicular to the gravity axis, i.e., along *y*. The sensor can be placed exemplarily on top or on bottom of the pipe (Figure 9). In the case that significant sedimentation occurred in the optically opaque slurry, the effective transverse relaxation will tend toward the values of water, while the bottom part will show faster effective transverse relaxation due to, among other effects, paramagnetic relaxation enhancement.

**Figure 10:**
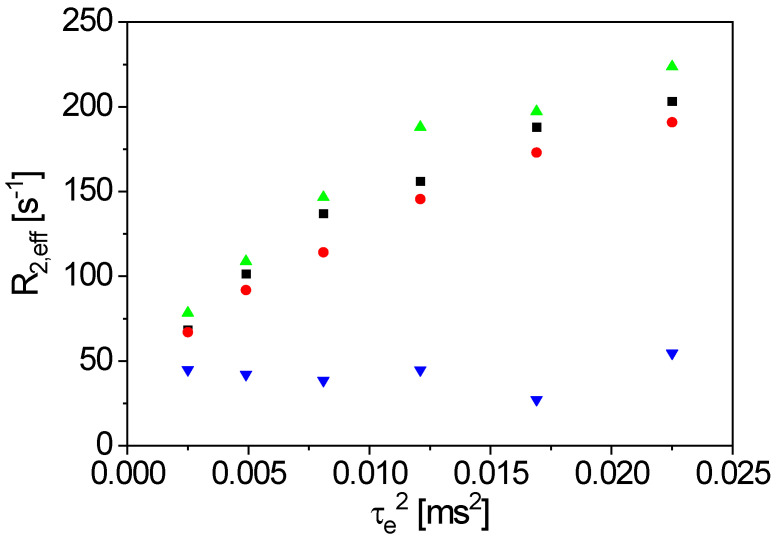
*R*_2,eff_(*τ_e_*^2^) for an anode slurry of 48.5%*w*/*w* graphite, 1%*w*/*w* CMC and 0.5%*w*/*w* carbon black, measured with the bent figure-8 coil on top and on the bottom of the horizontally fixed sample tube (■: bottom, 20 h; ●: top, 20 h; ▲: bottom, 7 days; ▼: top, 7 days. *R*_2,eff_ is larger at the bottom than at the top of the tube for both waiting times. *R*_2,eff_ is clearly larger at the bottom of the sample with a 7 day waiting time (▲), while value at the top (▼) approaches the values for doped water.

Thus, the anode slurry in a 10 mm pipe was fixed horizontally for 20 h to show whether sedimentation already occurs in the graphite slurry on that relatively short time scale. To investigate long-term effects, the sample was stored at the same position for seven days for a second measurement. CPMG measurements were carried out on the sedimented sample with the figure-8 coil at the bottom and on top of the sample tube, to analyze the specific areas inside the tube (Figure 9). *R*_2,eff_ for the two positions was measured for six values of *τ_e_* (Figure 10). 

The findings are in agreement with the observations described above: *R*_2,eff_ increases with *τ_e_* for the top and the bottom position in the first measurement (20 h). *R*_2,eff_ is smaller for all *τ_e_* at the top of the tube compared to the values at the bottom. This indicates the beginning of sedimentation due to the relaxation rates of “pure” water and water in the graphite sediment, containing paramagnetic impurities. Again, the measured points deviate slightly from a straight line, indicating sample heating. After a waiting time of seven days, the difference in relaxation between the position is even more evident: *R*_2,eff_ at the bottom position only slightly increased compared to the first measurement. *R*_2,eff_ lowered significantly at the top and is about constant with the small measured range of *τ_e_*. Sedimentation in the graphite slurry causes *R*_2,eff_ to approximate values at the top, similar to those measured on doped water. Please note again the small values of *τ_e_*, as significantly larger echo times would have to be measured to determine the diffusion coefficient from the slope. The lower *R*_2,eff_ results from a lower local concentration of graphite particles because of the longer sedimentation time. Nevertheless, the value of *R*_2,eff_ is larger than that for pure water due to dissolved ions from the graphite particles. The results indicate that sedimentation is an issue even in the slurries of technically relevant composition.

## 4. Conclusions

The inline-capable NMR sensor with its dedicated hardware configuration was characterized. Different coil geometries were compared with the finding that the bent figure-8 coil is the shape with optimal characteristics for the desired application from the perspective of quality control. Simulations were carried out and compared to the measurement results. The V-shaped NMR sensor, equipped with the bent figure-8 surface coil, has the potential to be used in quality control, for example in battery anode production, based on the relaxation and diffusion properties of the used materials. The application is not limited to graphite slurries but can be extended to other mixtures and disperse systems and enables both offline and mobile inline NMR measurements.

## Figures and Tables

**Table 1:** Comparison of the technical and NMR parameters for the three types of rf coils.

Parameter	Solenoidal Volume Coil	Spirally Wound, Bent Surface Coil	Bent Figure-8 Surface Coil
Diameter/Size along *x*	13 mm	39 mm	24 mm
Number of turns	19	11	8
Diameter of the insulated copper wire	0.45 mm	0.8 mm	0.45 mm
Quality factor of rf circuit	318	168	147
90° pulse duration (16 dB attenuation)	13 µs	30 µs	9 µs
Receiver dead time (RDT)	25 µs	10.9 µs	11 µs
Typical receiver gain for a 10 mm water sample	68 dB	86 dB	70 dB
Profile width along *x* (left and right points at 10% from maximum)	4.3 mm; 21.2 mm	3.8 mm; 23.8 mm	0.4 mm; 19.6 mm
Distance at half signal along *y*	n.a.	2.5 mm	3.5 mm
Picture of the coil	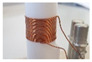	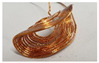	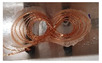

## Data Availability

The data are available on request to the authors.
